# Viewing geometry determines the contribution of binocular vision to the online control of grasping

**DOI:** 10.1007/s00221-017-5087-0

**Published:** 2017-09-12

**Authors:** Bruce D. Keefe, Simon J. Watt

**Affiliations:** 1 Department of Psychology, University of York, York, UK; 2 School of Psychology, Bangor University, Penrallt Rd., Bangor, Gwynedd LL57 2AS UK

**Keywords:** Grasping, Visual feedback, Binocular vision, Sensory integration, Online control, Visuo-motor control

## Abstract

Binocular vision is often assumed to make a specific, critical contribution to online visual control of grasping by providing precise information about the separation between digits and object. This account overlooks the ‘viewing geometry’ typically encountered in grasping, however. Separation of hand and object is rarely aligned precisely with the line of sight (the visual depth dimension), and analysis of the raw signals suggests that, for most other viewing angles, binocular feedback is less precise than monocular feedback. Thus, online grasp control relying selectively on binocular feedback would not be robust to natural changes in viewing geometry. Alternatively, sensory integration theory suggests that different signals contribute according to their relative precision, in which case the role of binocular feedback should depend on viewing geometry, rather than being ‘hard-wired’. We manipulated viewing geometry, and assessed the role of binocular feedback by measuring the effects on grasping of occluding one eye at movement onset. Loss of binocular feedback resulted in a significantly less extended final slow-movement phase when hand and object were separated primarily in the frontoparallel plane (where binocular information is relatively imprecise), compared to when they were separated primarily along the line of sight (where binocular information is relatively precise). Consistent with sensory integration theory, this suggests the role of binocular (and monocular) vision in online grasp control is not a fixed, ‘architectural’ property of the visuo-motor system, but arises instead from the interaction of viewer and situation, allowing robust online control across natural variations in viewing geometry.

**Electronic supplementary material:**

The online version of this article (doi:10.1007/s00221-017-5087-0) contains supplementary material, which is available to authorized users.

## Introduction

Efficient grasping requires fine online control of the position and closing speed of the digits with respect to a target object. Vision plays an important role in this process, and the contribution of binocular vision, specifically, is often considered critical (Sheedy et al. 1986; Morgan 1989; Previc 1990; Servos et al. 1992). At face value this seems logical: binocular vision (disparity and ocular convergence) can specify metric depth and, importantly for online control, disparity alone can provide precise information about the relative separation-in-depth of ‘objects’ (e.g., digits and target objects), and its rate of change (Gårding et al. 1995; Howard and Rogers 2002; Anderson and Bingham 2010). Consistent with this, and supported by evidence from neuropsychology, it has been proposed that there is a distinct “vision-for-action” system in the brain that relies selectively on binocular vision (Marotta et al. 1997; Goodale and Milner 2004). That is, there is a neural system dedicated to functions such as grasp control, which is hard-wired to rely on binocular input, with the brain “switching” to different neural systems (that also process monocular information) when binocular input is removed (Marotta et al. 1997). We refer to this as the ‘binocular specialism’ account (Keefe et al. 2011). Sensory integration theory offers a fundamentally different account. Here, the contribution of different signals to perception and visuo-motor control is determined by their informativeness in a given situation, relative to other available signals (Clark and Yuille 1990; Landy et al. 1995; Ghahramani et al. 1997; Ernst and Banks 2002). Thus, the contribution of binocular vision to online grasp control would be determined by (variable) factors that affect its informativeness, rather than being an architectural feature of underlying neural mechanisms for visuo-motor control. In this paper we examine whether the role of binocular vision in online grasp control is consistent with predictions of sensory integration theory, or is instead better accounted for by the binocular specialism account.

**Fig. 1:**
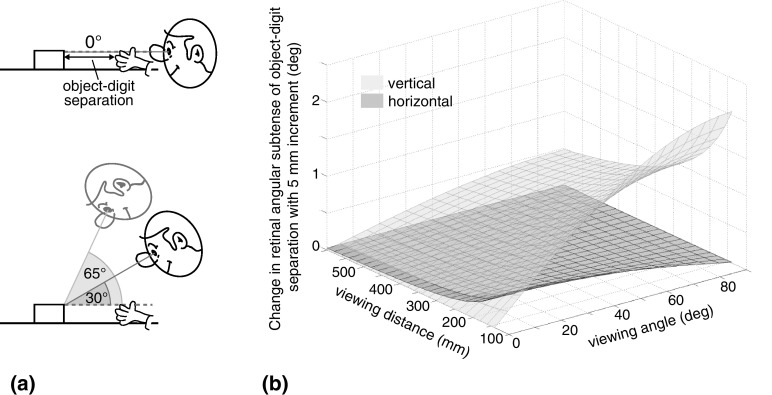
Effect of viewing geometry on the relative precision of binocular and monocular signals to object-digit separation. **a** Side view of grasping movements towards an object on a table, along the body midline, seen from different “viewing angles”—defined here as the angle between the line of sight (solid line) and the line along which digits and object are separated (dashed line; here, parallel to the table surface). **b** Angular change in object-digit separation at the retina, given a fixed (5 mm) increment in object-digit separation in the world, along the dashed line in panel a, as a function of distance and viewing angle. The dark-grey surface plots horizontal changes (the constituent signal for binocular disparity) and the dark-grey surface plots vertical changes (the monocular elevation signal). See “Appendix” for a detailed description of how these data were calculated

From the perspective of the visual information available for online grasp control, selective reliance on binocular vision seems unlikely to provide a robust general solution. The fundamental reason for this is that grasp control is often not solely a problem of controlling object–digit separation in the visual depth dimension (where binocular vision might be expected to be most useful). Consider the viewing geometry typically encountered in grasping. Even for movements straight ahead, along the body midline, only in the case of grasping objects exactly along the line of sight (top panel in Fig. 1a) does the separation of the digits and object correspond precisely to the visual depth dimension, specified by binocular disparity. Here, disparity must play a critical role because it is the principal informative signal (assuming monocular information from retinal size of the digits/object, and accommodation, is very imprecise; Fisher and Ciuffreda 1988; Bingham et al. 2001). But for other, more typical situations (imagine grasping a coffee cup on your desk) the eyes are positioned above the scene, and so the digits and object are separated not only in the depth dimension, but also in elevation in the visual array (bottom panel in Fig. 1a), providing potentially useful monocular signals for online control. Indeed, as the angle at which the hand and object are viewed increases the separation of digits and objects corresponds less and less to the visual depth dimension, and so disparity becomes less informative for this aspect of online grasp control, while monocular signals become more informative (see below). A system reliant solely on binocular input would perform poorly in these circumstances.

Sensory integration theory describes the ideal way to make use of multiple sensory signals that vary in informativeness. According to this account, the brain does not rely selectively on any one source (binocular vision, for example), but instead integrates information from all available signals, with more informative (more precise, or reliable) signals given more weight (Clark and Yuille 1990; Landy et al. 1995; Ghahramani et al. 1997). The precision of different signals depends strongly on scene-specific viewing parameters such as object distance and orientation with respect to the viewer (McKee et al. 1990; Knill 1998; Gepshtein and Banks 2003; Knill and Saunders 2003; Hillis et al. 2004; Takahashi et al. 2009; Keefe et al. 2011). There is now a body of work in depth perception and visuo-motor control indicating that the nervous system takes these changes into account, in a manner that is quantitatively very similar to the predictions of sensory integration theory (Knill and Saunders 2003; Hillis et al. 2004; Greenwald et al. 2005; Greenwald and Knill 2009a, b). This renders the nervous system robust to changes in signal precision by combining information to make best use of whatever signals are currently available.

Figure 1 explores in more detail the implications of sensory integration theory for the online control of grasping movements, seen from different viewpoints. The figure examines the relative precision of the raw binocular (disparity) and monocular (elevation) signals to object-digit separation, for various ‘viewing angles’. We consider grasping movements made along the body midline, towards an object on a table (Fig. 1a), for the typical experiment task (also common in the real world) of grasping an object front-to-back. That is, when the object is grasped, the grip aperture (a virtual line between finger and thumb) is parallel both to the body midline and the table surface. In a simplified situation, we considered the thumb and the near surface of an object as two points in space, separated in a direction parallel to the table surface. To characterise signal precision we first calculated the angle subtended at the retina by a given object-digit separation, for a range of viewing angles and distances. We then calculated the changes in angular subtense (separately for horizontal and vertical directions) that resulted from incrementing object-digit separation by a given amount (Fig. 1b; see “Appendix” for details). The resulting values describe the precision of the signals, in that they index the extent to which a change in object-digit separation in the world—in the direction of the table surface—is present in the retinal image, and so is available to the visual system. Horizontal changes (dark-grey surface) are the principal constituent signal for binocular disparity, and vertical changes (light-grey surface) form the monocular elevation signal. The absolute values in Fig. 1b are arbitrary, because they depend on the simulation parameters chosen. And this situation is of course much simpler than real movements, where (i) reach trajectories are typically curved above the table surface, rather than being precisely parallel to it, and (ii) both thumb and finger (and/or grip aperture) must be explicitly controlled (Jeannerod 1984; Smeets and Brenner 1999; Volcic and Domini 2016). Nonetheless, it allows relative comparisons to be made about how binocular and monocular retinal signals to object-digit separation vary in informativeness as the direction of separation varies with respect to the line of sight. There are clear effects of viewing geometry. As described above, grasping along the line of sight (viewing angle = 0°) results in a signal only in the horizontal direction, and so binocular disparity must play a critical role in this situation. However, the precision of the horizontal signal (from which disparities are computed) exceeds the vertical (monocular) signal only for a very small range of viewing angles beyond which vertical shifts dominate (as a reference, an informal survey of colleagues’ offices suggests viewing angles of around 40°–60° are typical for a seated person grasping the apocryphal coffee cup). This remains the case even at small viewing distances, where the precision of the disparity signal improves non-linearly (Hillis et al. 2004). Thus, sensory integration theory predicts that the contribution of binocular vision to online grasp control will decrease systematically with increasing viewing angle.

We report the results of an experiment examining whether the role of binocular vision in online grasp control varies with viewing angle, in the manner predicted by sensory integration theory, or is instead consistent with the binocular specialism account. Following previous work, we assessed the contribution of binocular vision to online grasp control by measuring the effect on grasp kinematics of covering one eye at movement onset. We did this at three different viewing angles, to vary the relative precision of binocular and monocular signals to digit-object separation. According to sensory integration theory, the contribution of binocular vision to online grasp control will decrease systematically with increasing viewing angle, as the relative precision of the binocular signal is reduced (Fig. 1). Loss of information that contributes to online control should be evident primarily in the later phases of grasping movements, in particular as an extended final slow-movement phase as the digits close on the object (Servos and Goodale 1994; Jackson et al. 1997). Sensory integration theory therefore predicts that removing binocular feedback will result in systematically smaller increases in time-in-the-slow-phase as viewing angle increases. Moreover, at large viewing angles (looking down upon the hand/object) binocular depth cues are considerably less informative about the digit-object separation (Fig. 1) and so their removal would be expected to have no substantive effect on the final slow-phase of movements. In contrast, the binocular specialism account proposes that changes in performance result not from loss of information per se but from disruption to a dedicated visuo-motor subsystem, built to receive binocular input. That is, performance is impaired due to the loss of a normal input ‘channel’, necessitating switching to a different neural system that is not specialised for visuo-motor control (Marotta et al. 1997; Goodale and Milner 2004). Specific predictions for varying viewing angle are unclear under this model, but there should be no situation in which removing binocular feedback does not impact online control. This account, therefore, predicts increased time in the slow phase at all viewing angles, including when looking directly down on the hand and object.

## Methods

### Participants

Sixteen right-handed participants took part in the experiment (6 female, 10 male, aged 21–45-years-old). All had normal or corrected to normal vision and stereoacuity better than 40 arcsec. None reported any motor deficits. Participants gave informed consent and were paid for their participation. The procedures were approved by the Ethics Committee of the School of Psychology, Bangor University, and were in accordance with the Declaration of Helsinki.

### Apparatus and stimuli

**Fig. 2:**
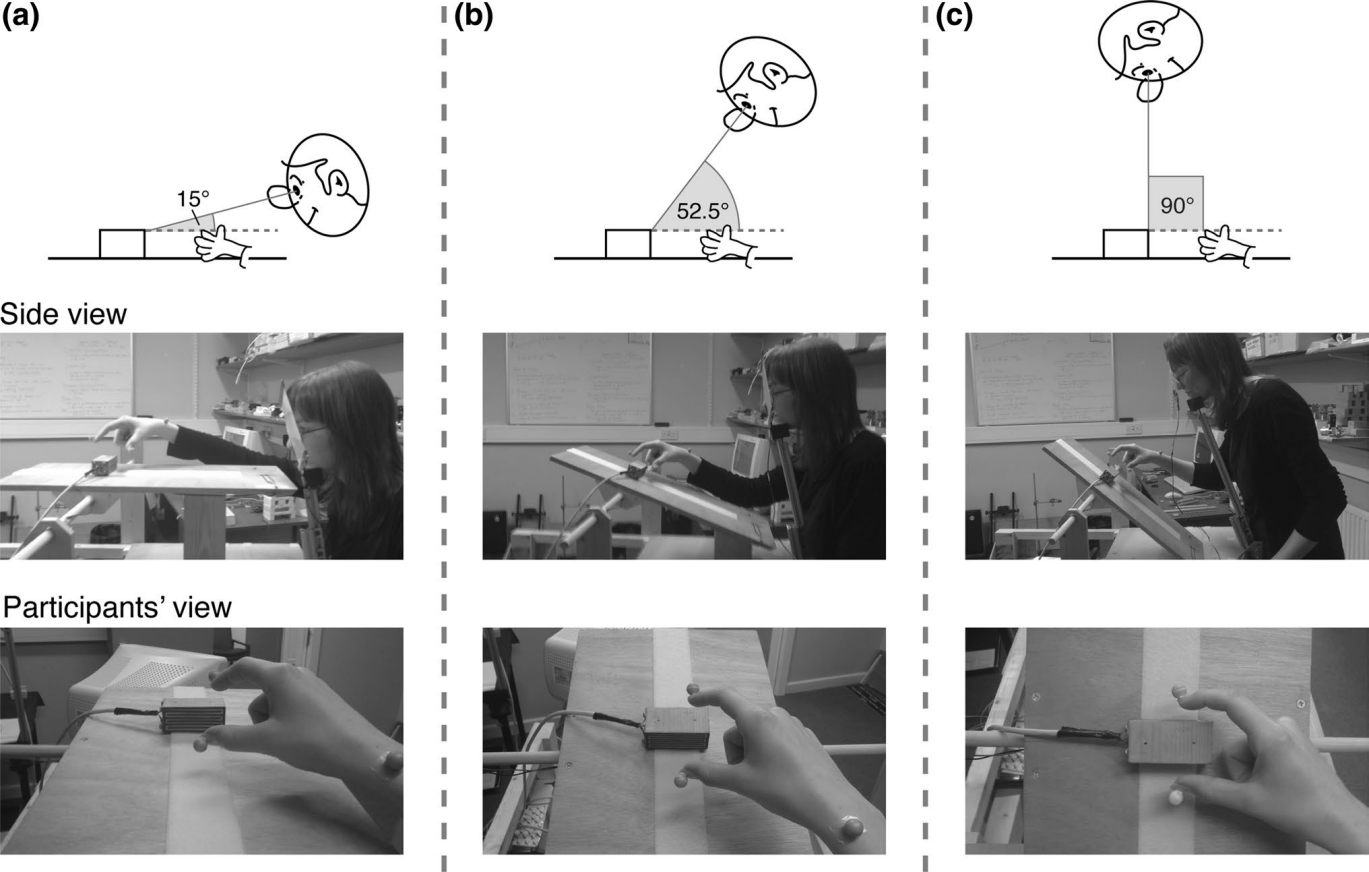
The three viewing-angle conditions. **a** The 15° condition in which the digits and object were separated primarily in the depth dimension. **b** The 52.5° condition in which the digits and object were separated in both the depth dimension and in the frontoparallel plane. **c** The 90° condition in which the hand and object were separated primarily in the frontoparallel plane. The top row illustrates the viewing geometry in each case in schematic form. The middle row shows how each condition was implemented in practice, by appropriate positioning of both the participant, and (moveable) table surface. The bottom row shows the participants’ view of the object and hand. Note that the photographs were staged to illustrate the experiment setup, and do not accurately represent the participants’ actual movement trajectories in the experiment (which are shown in the online Supplementary Material, Fig. 7)

We manipulated the relative precision of binocular and monocular feedback by varying the angle from which the object and hand were viewed (Fig. 2). Three different viewing angles—defined as the angle between the direction of digit-object separation and the line of sight—were used: 15°, 52.5° and 90° (Fig. 2, top row). Because it was not practical for participants to lean over the table for a whole experimental block, we varied viewing angle using a combination of moving the participants’ head position, and rotating the “ground plane” (Fig. 2, middle row). For all viewing angles, the required trajectory of the hand was similar with respect to the ground plane (grasping movements began at a start button on the ground plane surface). An analysis showing that actual movement trajectories were similar at all viewing angles is presented in Supplementary Material (Fig. 7). The bottom row in Fig. 2 shows the participants’ view of the hand and object in each case. An adjustable chin rest was used to maintain eye/head position. We positioned participants using a bespoke sighting device, allowing us to minimise vertical (and lateral) errors in viewing position. By way of comparison, we estimate that the typical viewing angle used in previous studies was around 40°–55° (Bradshaw and Elliott 2003, provided precise information, but head position is typically uncontrolled). Thus, our 52.5° condition conforms most closely to previous work (Servos and Goodale 1994; Jackson et al. 1997; Bradshaw and Elliott 2003).

To manipulate visual feedback, participants wore plain-lens spectacles (zero optical power) with a piece of Liquid Crystal “smart glass” film covering the left eye (visible in Fig. 2; PolyVisionTM, United Kingdom). The film changed from opaque to transparent when a current was applied. We either occluded the left eye on detection of movement onset (monocular visual feedback condition) or left the film transparent (binocular visual feedback condition). In all conditions the grasping hand was visible throughout the whole movement.

Participants grasped three object sizes, at three movement distances, to vary the required movement trial-by-trial. The objects were wooden blocks, with depths of 25, 35 and 45 mm (i.e., front-to-back; the grasped dimension). They were 70 mm wide and 30 mm tall. The objects were lightly attached to the ground plane using fabric hook-and-loop fastener so they could be lifted easily, but did not move when the ground plane was rotated from horizontal. Objects were presented at distances of 300, 350 and 400 mm from the start switch (measured along the ground plane) on the body midline. In all viewing-angle conditions, objects at the middle distance (350 mm) were 450 mm from the subject’s cyclopean eye. The different viewing geometries resulted in slightly different average viewing distances across conditions, but these differences were very small (<3 mm).

The front and rear contact surfaces of the objects were each covered with two interlocking patterns of conductive fabric (visible in the bottom-left panel of Fig. 2; Schlegel Electronic Material, Inc., Belgium), forming an open circuit. When the surface was touched, skin conductance closed the circuit, allowing us to record the time at which the thumb and finger grasped the object. The precise force required to trigger the sensors varied with skin conductance and finger/thumb pad area, but separate tests showed that object contact was typically detected at grip forces of ~0.5 to 1.0 N, which, for our objects, equates to a static grip-force to load-force ratio of ~1.2 to 2.3. Thus, the sensors were triggered only by relatively firm contact between digits and surfaces (not by merely brushing against them) corresponding to the acquisition of stable grasp points (Hiramatsu et al. 2015).

Grasping movements were recorded using a ProReflex infrared motion capture system (Qualisys AB, Sweden). The system captured the *x*, *y*, *z* positions of markers attached to the nails of the thumb and index finger, and to the skin of the wrist (Fig. 2, bottom row), at 240 Hz.

### Procedure

At the start of each trial the room was completely dark and the participant pressed the start switch with his or her right thumb and index finger, pinched together. A trial was initiated by switching on a table lamp (controlled by the experiment computer), to illuminate the scene. Participants viewed the object binocularly for 2 s, after which an audible beep sounded indicating they should grasp the object with their right hand. Participants were instructed to pick up the object quickly and naturally, front-to-back, using only their thumb and index finger. On binocular feedback trials the left eye’s view remained unoccluded for the whole trial. On monocular feedback trials the left eye was occluded at movement onset. Movements that began before the start signal, or more than 600 ms after it, were considered void and repeated at the end of the block. The number of “void trials” equated to ~5% of the total, and was independent of viewing angle and feedback condition. The lamp remained on throughout the movement except in void trials, where it was extinguished at movement onset. The lamp was turned off between trials and participants closed their eyes while the experimenter positioned the target object using a small torch/flashlight.

The experiment was blocked by both the type of feedback (binocular or monocular) and viewing angle (15°, 52.5° or 90°). Blocking by viewing angle was necessary because it was not practical to reposition the participant trial-by-trial. We blocked trials by feedback type because trial-by-trial randomising of visual feedback conditions can result in movements that are planned to anticipate having the poorest feedback on all trials (Jakobson and Goodale 1991). Each participant completed six repetitions of each stimulus combination (object × distance × viewing angle × feedback condition = 324 trials). Within a block, object sizes and distances were randomly ordered, and condition order was counterbalanced.

### Movement indices

For each trial, the 3D co-ordinates of each marker were low-pass filtered (Butterworth filter, 12 Hz cut-off) before computing movement trajectories. From these data, together with finger/thumb contact times, we then characterised each movement by computing: (1) peak wrist velocity of the movement; (2) peak grip aperture (the maximum separation between the pad of the thumb and index finger, corrected for marker location); (3) time in the slow phase—the final slow-movement phase as the hand closes in on the object, defined as the time elapsed from the point at which peak wrist deceleration occurred to when the last digit (thumb or finger) made stable contact with the object (see “Apparatus and stimuli”).

### Predictions

Our experiment was designed to isolate, and answer a specific a priori question about, one particular aspect of online control. We therefore, constrained our analyses to testing a small number of well-specified, directional predictions, for key dependent measures, rather than carrying out global analyses. Moreover, while the required movements at different viewing angles were broadly similar, they necessarily could not be identical because shoulder position varies with head position (and because we manipulated the table surface orientation). We therefore, determined the effect of removing binocular feedback within each viewing angle, and then compared these effects across viewing angles, as opposed to making direct, absolute comparisons of, say, binocular performance across viewing angles.

Although perturbation studies have revealed that visual feedback is used throughout arm and hand movements (e.g., Saunders and Knill 2003; Fukui and Inui 2015; Chen and Saunders 2016), manipulations of the availability of feedback during grasping primarily affect the later stages of the movement (Jakobson and Goodale 1991). In this situation, it makes sense that the influence of feedback becomes more evident the longer the movement progresses. Moreover, feedback arguably plays a more critical role later in grasping movements, where precise control of the final ‘landing’ of the digits on the object’s surfaces is paramount. Binocular feedback is also in principle most precise—and so most useful—at the end of the movement when the digits and object surfaces are relatively close together, appearing simultaneously in the fovea, and creating relatively small binocular disparities that do not exceed fusional limits (see Banks et al. 2004). Consistent with this, loss of binocular feedback at movement onset has previously been found to cause prolonged time in the slow phase, suggesting a more “cautious” approach to the object when information about the relative position of digits and object surfaces is degraded/unavailable (Servos and Goodale 1994; Jackson et al. 1997; Bradshaw and Elliott 2003). Time in the slow phase was, therefore, our principal dependent measure. A larger contribution of binocular vision to online control should be evident as a larger increase in time in the slow phase when binocular feedback is unavailable. Thus, if this contribution depends on viewing geometry, as predicted by sensory integration theory and the analysis in Fig. 1, removing binocular vision will have the largest effect at small viewing angles (grasping near to the line of sight), decreasing to little or no effect as viewing angle increases (looking down on the digits and object). The binocular specialism account does not make specific predictions for effects of viewing angle, but predicts that loss of binocular feedback will disrupt functioning of neural systems underpinning grasping in all cases, resulting in longer time in the slow phase even at the largest viewing angle.

It is difficult to make predictions regarding whether or not kinematic indices that occur earlier (e.g., peak wrist velocity and peak grip aperture) would be demonstrably affected by removal of binocular feedback. Indeed, empirical results from previous studies that are most similar to ours provide an unclear picture. Both Jackson et al. (1997; Expt. 3), and Bradshaw and Elliott (2003), found increased peak grip apertures when binocular feedback was removed at movement onset. Servos and Goodale (1994; Expt. 1) found no effects on grip aperture with the same manipulation, however, and none of these studies found significant effects of removing binocular feedback on peak wrist velocity. Nonetheless, it is reasonable to expect that loss of useful information from binocular vision would, if anything, result in larger peak grip apertures and reduced peak wrist velocities (Jackson et al. 1997; Watt and Bradshaw 2000; Loftus et al. 2004; Melmoth and Grant 2006; Keefe and Watt 2009). Moreover, we considered it important to analyse these indices anyway, because they provide a general characterisation of the movements, allowing us to determine if normal, stereotypical properties were present in all conditions (scaling of peak velocities with object distance, and scaling of peak grip apertures with object size; Jeannerod 1984, 1988). This was of particular importance here because of the unusual situation of a non-horizontal table surface in some conditions.

Because we had specific, directional predictions we evaluated statistical significance by making planned pairwise comparisons. For each dependent measure, binocular vs. monocular comparisons were used to test for a contribution of binocular feedback within each viewing-angle condition. Viewing angle dependence was examined by comparing the effect of removing binocular information at the smallest vs. largest viewing angles (15° vs. 90°).

## Results

### Time in the slow phase

**Fig. 3:**
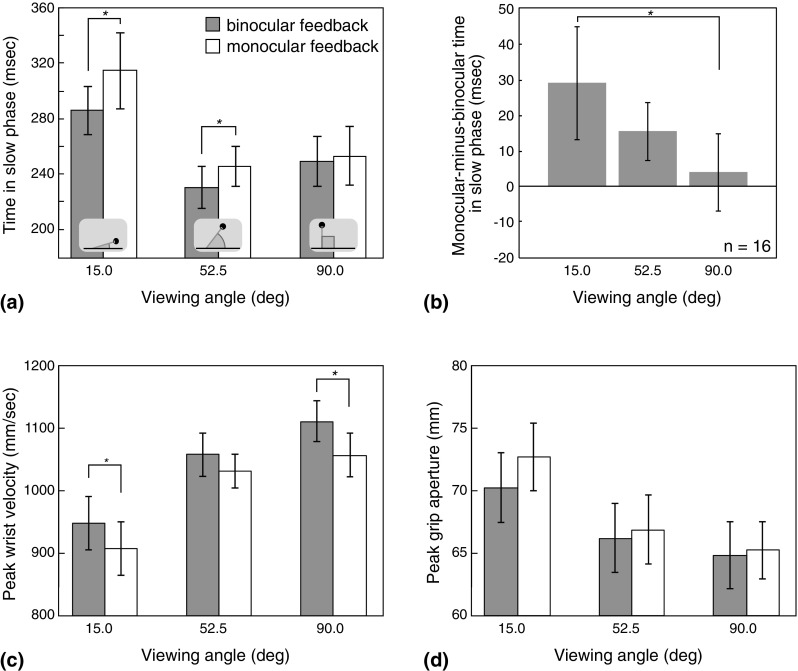
Main experiment results. **a** Average time in the slow phase at each viewing angle. Dark bars denote binocular feedback conditions and light bars denote monocular feedback. **b** Average effect of removing binocular feedback on time in the slow phase (monocular−binocular) at each viewing angle. **c** Average peak wrist velocity for each viewing angle and feedback condition. **d** Peak grip aperture for each viewing angle and feedback condition. In all plots, error bars denote ± 1 SEM. Asterisks denote statistically significant pairwise comparisons (see main text and Table 1)

Figure 3a plots the overall mean (*n* = 16) time in the slow phase, averaged across object size and distance, for each feedback type and viewing angle. In line with predictions of sensory integration theory, the figures show a systematic effect of viewing angle on the contribution of binocular information to this phase of the movement. At the 90° viewing angle (looking down on the object and hand) time in the slow phase was essentially the same with or without binocular feedback. As viewing angle decreased, however, removing binocular feedback resulted in relatively longer time in the slow phase. Table 1 reports planned pairwise comparisons between time in the slow phase with monocular vs. binocular feedback, at each viewing angle (see “Predictions”). Removing binocular feedback resulted in significantly longer time in the slow phase at viewing angles of 15° and 52.5°, but not at 90°.

**Table 1:** Statistical effects of removing binocular feedback

Dependent measure	Viewing angle		
	15°	52.5°	90°
Time in the slow phase	*t* = 1.83, *p* = 0.044*	*t* = 1.89, *p* = 0.039*	*t* = 0.36, *p* = 0.363
Peak wrist velocity	*t* = 2.17, *p* = 0.023*	*t* = 1.21, *p* = 0.122	*t* = 2.15, *p* = 0.024*
Peak grip aperture	*t* = 1.53, *p* = 0.073	*t* = 0.57, *p* = 0.287	*t* = 0.30, *p* = 0.384

Planned *t* tests (*df* = 15; one-tailed) between binocular and monocular feedback conditions, for each viewing angle (collapsed across object size and distance)

Asterisks denote statistically significant pair-wise comparisons

To analyse directly how the contribution of binocular feedback depended on viewing angle, we compared the effect of removing binocular feedback at different viewing angles. Figure 3b plots the difference between each participant’s average time in the slow phase in binocular and monocular feedback conditions, collapsed across object distance and size (monocular minus binocular, so positive values indicate longer time in the slow phase with monocular feedback), averaged across all observers. It can be seen that increasing viewing angle (increasingly looking down on the hand and object) resulted in systematically smaller effects of removing binocular feedback. Consistent with this, pairwise comparison of the difference scores showed that the effect of removing binocular feedback was significantly larger at the 15° viewing angle than at 90° (one-tailed *t* test; *t* = 2.20, *p* = 0.026). These results are consistent with the predictions of sensory integration theory regarding the usefulness of binocular feedback at different viewing angles. They suggest that binocular feedback played little role in feedback control when the hand and object were primarily separated in the frontoparallel plane, but made an increasing contribution as the direction of object-hand separation approached the visual depth dimension. The lack of any effect of the loss of binocular feedback on time in the slow phase at the 90° viewing angle is inconsistent with disruption to a binocular-only grasping system (due to loss of a normal input channel).

### Peak wrist velocity

We examined peak wrist velocity in each condition as a function of object distance, and confirmed that the data conformed to the stereotypical pattern of increased velocity with increasing object distance (Jeannerod 1984, 1988) in all conditions, including those where the table surface was not horizontal. These data are presented in the Supplementary Material (Fig. 5).

Figure 3c plots average peak wrist velocity for binocular and monocular feedback conditions for each viewing angle, collapsed across object size and distance. It can be seen that movements were generally slightly slower with monocular feedback, but that there was no obvious dependence on viewing angle. Table 1 reports planned pairwise comparisons between peak wrist velocity with monocular vs. binocular feedback, at each viewing angle (see “Predictions”). These tests indicated that movements were significantly slower with monocular feedback in both the 15° and 90° viewing-angle conditions, but not the 52.5° condition. As with time in the slow phase, we evaluated the effect of viewing angle by conducting a *t* test comparing binocular-minus-monocular difference scores at 15° vs. 90° conditions (see “Predictions”). The result was not significant (*t* = 0.50, *p* = 0.314, one-tailed). Thus, while movements were slower overall with monocular feedback for two of the three viewing angles, these effects did not depend systematically on viewing angle.

### Peak grip aperture

Similar to above, we examined peak grip aperture in each condition as a function of object size, and confirmed that the data exhibited the stereotypical pattern of increased peak grip aperture with increasing object size (Jeannerod 1984, 1988) in all conditions. Again, these data are presented in the Supplementary Material (Fig. 6).

Figure 3d plots average peak grip apertures for binocular and monocular feedback conditions for each viewing angle (collapsed across object size and distance). Table 1 shows that removing binocular feedback did not have a statistically significant effect at any of the viewing angles. Figure 3d is suggestive of a systematic effect of viewing angle: the largest effect of removing binocular feedback occurred at the 15° viewing angle, consistent with binocular feedback making a larger contribution as viewing angle decreases. However, analysis of binocular-minus-monocular difference scores (as above) showed that the effects of removing binocular feedback at 15° and 90° viewing angles were not significantly different (one-tailed *t* test; *t* = 0.71, *p* = 0.245).

## Discussion

We tested the prediction that the contribution of binocular vision to online control of grasping depends on the “viewing geometry” of the separation between digits and object, as opposed to being an architectural feature of neural mechanisms for visuo-motor control. Our study was motivated by two observations. First, for geometrical reasons, binocular disparity might be expected to be the most precise visual signal for online control only when digits and object are separated primarily along the line of sight. For many other viewing angles monocular signals are potentially more precise, and so a system built to rely selectively on binocular vision offers a poor general solution. Second, there is growing evidence that, as a general rule, the visuo-motor system integrates all available signals, weighting them according to their precision (reliability) in a particular situation. Taken together, these observations predict that the contribution of binocular vision to online grasp control will decrease as subjects increasingly look down on the separation between object and moving hand. This is what we found. The effect of removing binocular feedback (increased time in the final slow-movement phase, as the digits approached the object; Servos and Goodale 1994; Jackson et al. 1997; Bradshaw and Elliott 2003) was greater when the digits and object were separated primarily along the line of sight, compared to when they were separated primarily in the frontoparallel plane. This indicates that binocular vision played a significantly smaller role in the latter case. Indeed, this final phase of movement was unaffected by loss of binocular feedback when the digits and object were separated in the frontoparallel plane.

Our findings are consistent with the idea that online visual control of precision grasping obeys the general principles of sensory integration theory. That is, the available signals are combined, with the contribution, or “weight”, of each determined by its precision (reliability) in a particular circumstance. Because the precision of different signals depends differently on “geometrical factors” such as distance and orientation with respect to the head, viewing geometry is a determinant of their relative precision, and therefore contributions, moment-by-moment, allowing online grasp control to be robust to changing viewing parameters. Our study does not speak to the specific, quantitative question of whether or not grasp control signals are integrated in a statistically optimal fashion in online grasp control. Nonetheless, it adds to an emerging consensus that the principle of reliability-based signal weighting applies generally to situations in which information is available from binocular and monocular vision, both for perception (e.g., Knill and Saunders 2003; Hillis et al. 2004) and visuo-motor control (e.g., Knill 2005; Greenwald et al. 2005; Greenwald and Knill 2009a, b; van Mierlo et al. 2009; Keefe et al. 2011).

An important emergent principle from a sensory integration account of online grasp control is that the role of binocular (and monocular) signals arises from the interaction between the visual system and the particular context, rather than being a fixed feature of the visuo-motor system. As such, this account argues against the long-standing idea that binocular vision has a special or privileged role in visuo-motor control. The most prominent of such proposals, discussed earlier, is the idea of a structurally distinct neural system for vision-for-action that is hard-wired to rely on binocular vision (Marotta et al. 1997; Goodale and Milner 2004). A more specific, and differently motivated, idea is that online grasp control is achieved simply by nulling the binocular disparity between the digits and object surfaces (Morgan 1989; Mon-Williams and Dijkerman 1999; Bradshaw and Elliott 2003; Anderson and Bingham 2010). This idea has appealing simplicity, in that the control process could operate directly on retinal signals without the need for constructing internal representations of metric properties such as digit-object separation distance (Anderson and Bingham 2010). But a strict interpretation of this proposal too—that online grasp control relies exclusively on binocular disparity—is not supported by our data, or other empirical evidence that binocular and monocular signals are used together (i.e., integrated) in visuo-motor control. Moreover, as discussed in the “Introduction” consideration of the informativeness of binocular signals with different viewing geometries highlights how, in principle, selective reliance on binocular disparity would not be a robust, effective strategy across naturally occurring changes in viewing circumstances (McKee et al. 1990; Knill 1998; Gepshtein and Banks 2003; Knill and Saunders 2003; Hillis et al. 2004; Takahashi et al. 2009; Keefe et al. 2011).

Of course situations may nonetheless arise in which binocular vision plays the major, or even a critical, role in online control (when monocular information is very unreliable; Fig. 1; Read et al. 2013). We suggest, however, that these should be thought of as specific instances of a general mechanism of sensory integration (a weight of near 1.0 given to binocular information), rather than indicating an inherent specialism for binocular vision. In this context, note that changes in signal weights (including dominance by one signal) do not imply connectivity changes, or plasticity, in the underlying neural mechanisms, but merely the processing of different information by a common integration mechanism (Knill and Pouget 2004; Natarajan and Zemel 2011; Fetsch et al. 2012).

Our study was not designed to distinguish between different accounts of the specific “control mode” via which visual information is used in visual grasp control; whether grasp aperture, or the separation between digits and target locations is explicitly controlled, for example (Jeannerod 1984; Smeets and Brenner 1999; Volcic and Domini 2016). This factor could in principle interact with our manipulation of viewing angle. Volcic and Domini (2016), for example, recently presented evidence that as the viewing angle increases—and so the far edge of the target object (and the finger) is increasingly visible—online control contributes more to control of grip aperture per se, and less to guiding the thumb to the (visible) near surface of the object. This raises the possibility that not only does the relative precision of different signals change with changes in viewing angle, but also that the underlying parameters that are controlled in grasping may change too. Note, however, that the analysis of signal precision in Fig. 1 applies generally to visual information about the separation of any two objects with respect to viewing angle, including the finger and thumb. Thus, for grasping movements such as those examined here, changes in viewing angle would affect the relative precision of binocular and monocular signals to grip aperture in a similar manner to signals to digit-object separation. The relative contribution of binocular vision to controlling either parameter—grip aperture, or digit trajectory/object-digit separation—would, therefore, be expected to be affected similarly by viewing angle.

Our manipulation of viewing angle is based on the premise that hand movement trajectories were the same, independent of viewing condition. This is not a given for at least two reasons. First, our manipulation of viewing angle necessitated physically different arm movements, due to changes in shoulder position and table surface orientation. Second, it is possible that participants systematically altered their reach trajectories in different viewing-angle conditions, so as to always move the hand in the same manner relative to the line of sight, effectively undoing the manipulation of viewing angle. To examine whether this was the case, we analysed average movement trajectories of the thumb in all conditions to see if they varied with viewing angle (and availability of binocular feedback). The data are presented in the online Supplementary Material (Fig. 7). This analysis showed that the thumb movement trajectories were largely similar (with respect to the table surface) across all conditions, confirming that our manipulation did result in a change of viewing angle of the moving hand. The average direction of thumb travel over the last half of the movement was not parallel to the table, but was reasonably closely aligned with the line of sight in the 15° viewing-angle conditions. For the final portion of the movement (the last ~7% of the movement distance) average thumb trajectories were closely aligned with the line of sight at 52.5° viewing angle. Thus, in our 52.5° condition—similar to typical viewing angles in previous studies—the effective viewing angle for this final phase of the movement was in fact much closer to 0°. This may explain why we, and others, observed significant effects of removing binocular vision in these circumstances, despite the very low relative precision of binocular information suggested by Fig. 1 (Servos and Goodale 1994; Jackson et al. 1997; Bradshaw and Elliott 2003). Moreover, the analysis confirmed that in the 90° viewing-angle condition, digits and object were separated not only in depth, but also vertically in the visual array (providing a monocular signal), even in the final phase of the movements. That is, participants did not alter their grasp trajectories to align with the visual depth dimension.

The effects of removing binocular feedback in our study were modest, even in conditions where it was most informative. Removing binocular feedback at the 15°, 52.5° and 90° viewing angles caused time in the slow phase to increase by 10.0, 6.7 and 1.5% (non-significant), respectively. At one level, this provides further support for the argument that binocular information is not critical for grasp control. Interpretations of the absolute value of these effects should be carefully qualified, however. Previous studies report somewhat larger effects than comparable conditions in our study. Bradshaw and Elliott (2003) used a viewing angle of ~40°, at similar viewing distances to ours (average = ~450 mm), and found that removing binocular feedback resulted in an 11% increase in deceleration time (time after peak wrist velocity). As mentioned previously, the viewing angle and distance are not known for the studies by Servos and Goodale (1994), and Jackson et al. (1997), but assuming a ‘typical’ viewing angle of 40°–60°, their effects of 11% increase in time in the slow phase, and 12% increase in deceleration time, respectively, are larger than the comparable effect we observed at 52.5° viewing angle. It is possible that we observed smaller effects in our study because we used a considerably larger number of trials (mostly due to having three viewing-angle conditions). This may have facilitated learning of stimulus properties and/or the required movement parameters, leading to less reliance on visual feedback overall (Keefe and Watt 2009; Fukui and Inui 2015). Consistent with this, movements in the fully binocular conditions in our study were also faster overall compared to those cited above, with ~28% higher average peak velocities (Servos and Goodale 1994; Bradshaw and Elliott 2003) and ~37% shorter time in the slow phase (Servos and Goodale 1994), for grasps made under comparable conditions. Moreover, in common with almost all lab-based studies, we significantly constrained the variability in the required movements by always positioning objects on the body midline, along a planar table surface, and using rectangular cuboid objects grasped in the same orientation. Many (though by no means all) real world situations involve grasping novel objects, in a less constrained manner (picking fruit from a tree, for example, where the height of the hand with respect to the object must also be visually controlled), in which case binocular vision may make a larger contribution. Furthermore, in the real world the relative precision of signals will depend not only on viewing geometry but also on other factors specific to the particular context, such as surface texture of the target object, presence of occluding objects and so on. Thus, quantitative conclusions about real world grasping should not be drawn from the absolute sizes of the effects in our study, or other similar studies. Indeed, in natural grasping people can also control viewing geometry by altering their head position and grasp trajectory. A complete account of signal use in grasp control therefore requires both measurement of the natural distribution of viewing geometries encountered in the real world, and exploration of whether viewing angle is actively controlled (e.g., with respect to varying signal precision). Our data nonetheless provide proof-of-principle evidence that viewing geometry modulates the relative contribution of binocular vision to online grasp control.

In summary, we have shown that changing viewing angle alters the contribution of binocular vision to online control of grasping in a manner consistent with changes in the relative precision of binocular and monocular information about the separation of the digits and target object. This result is consistent with sensory integration models proposing that the contribution of all available signals depends on their relative informativeness (reliability or precision) in a given context, allowing online grasp control to be robust across changing viewing situations. It is inconsistent with models proposing that the visuo-motor system relies selectively or preferentially on information from binocular vision. We suggest that the role of binocular vision in online grasp control should, therefore, be thought of as arising from the interaction of viewer and situation, not as a feature of the visuo-motor system per se.

### Electronic supplementary material

Below is the link to the electronic supplementary material.

Supplementary material 1 (DOCX 385 kb)

## Figures and Tables

**Fig. 4:**
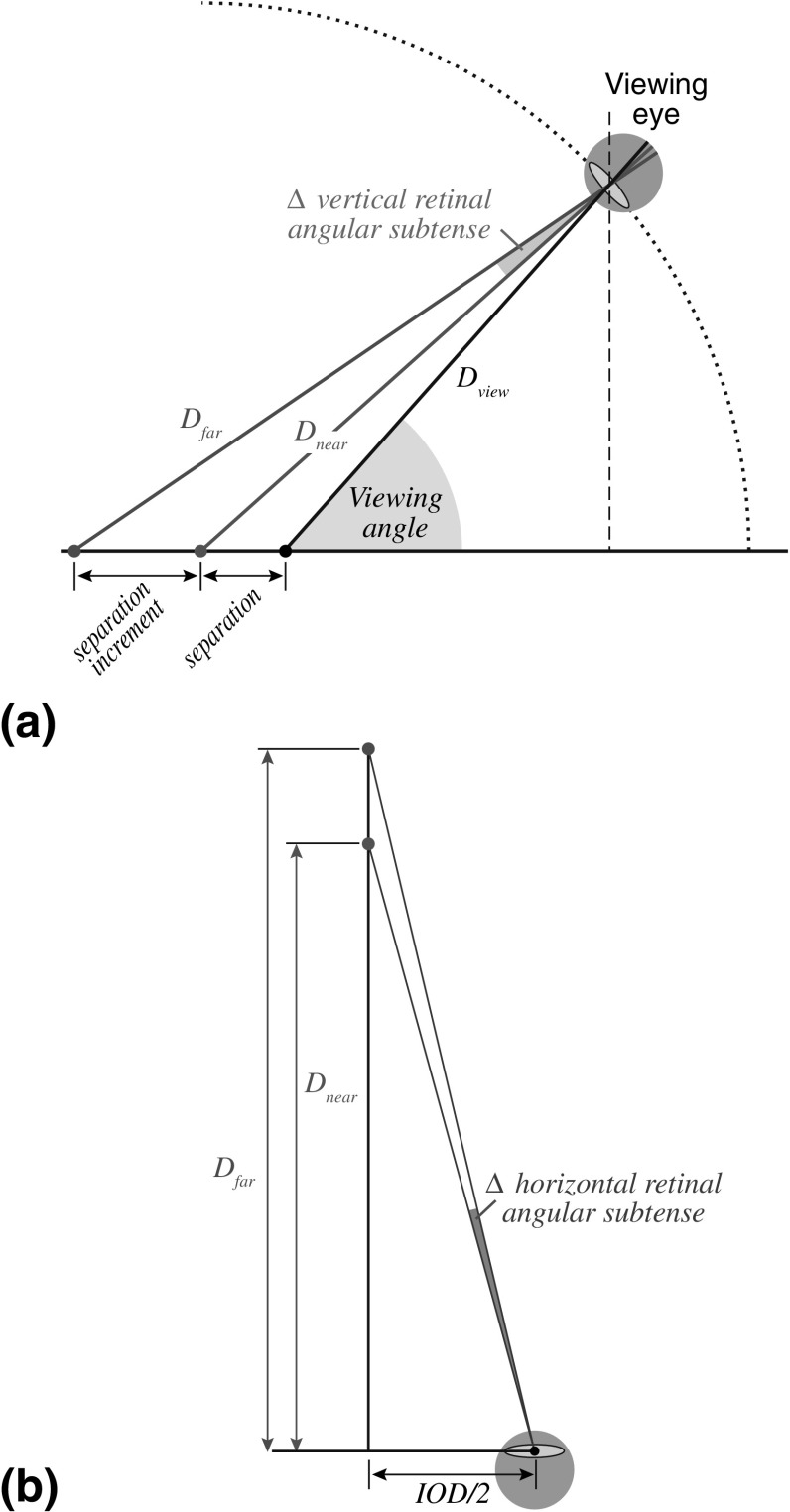
Geometry underlying relative signal precision calculation. **a** For vertical retinal shifts we used simple trigonometry to compute the change in angle subtended by a given separation of two points (∆vertical retinal angular subtense) given a 5 mm increment in their separation, along the horizontal plane. We did this for a range of viewing distances (*D*_view_) and viewing angles. It can be appreciated from Fig. 4a that, all else being equal, for small initial separations, ∆vertical retinal angular subtense depends principally on the separation increment, and not on the separation magnitude itself. We therefore simply analysed the angle subtended by the separation increment in each case (i.e., assuming zero initial separation of the points). **b** Horizontal retinal shifts (*∆*horizontal retinal angular subtense) are determined by the relative distances (*D*_near_ and *D*_far_) of the two points from the participant’s cyclopean eye (the virtual point midway between the two eyes), and the lateral position of the eye. *D*_near_ and *D*_far_ were derived trigonometrically (see **a**) and the lateral position of the eye was assumed to equal the interocular distance (IOD; here assumed to be 62 mm) divided by 2. Again, simple trignometry then yielded *∆*horizontal retinal angular subtense

## Appendix: Calculating the effect of viewing geometry on the relative precision of binocular and monocular signals to object-digit separation

Consider a situation in which a given change in position in the world results in a relatively large change in retinal position versus one in which the same change results in a small retinal change. Assuming fixed noise in sensing retinal position, the latter case must represent a less precise signal to change in position in the world. We therefore analysed relative precision of horizontal and vertical signals to object-digit separation by considering the change at the retina caused by a given change in separation in the world, at a range of viewing angles and distances. For simplicity, we treated the thumb and near surface of the object as two points in space, separated along a line parallel both to the body midline, and to a horizontal table surface. Figure 4 shows the geometry used to calculate signal precision in each case. Further details are provided in the figure caption.
