# Supplementary material for: Viewing geometry determines the contribution of binocular vision to the online control of grasping
Source: Exp Brain Res. 2017 Sep 12;235(12):3631–43. doi: 10.1007/s00221-017-5087-0 (PMC5671520; doi:10.1007/s00221-017-5087-0)
Supplement: Supplementary file 1 — Supplementary material 1 (DOCX 385 kb) [file 221_2017_5087_MOESM1_ESM.docx]

**Viewing geometry determines the contribution of binocular vision to the online control of grasping.**

Bruce D. Keefe^1^ and Simon J. Watt^2^

^1^ Department of Psychology, University of York, York, UK

^2^ School of Psychology, Bangor University, Bangor, UK

**PEAK WRIST VELOCITY ‘SCALING’**

Figure 5 plots mean peak wrist velocity in each condition, as a function of object distance (collapsed across object size). Panels a-c plot binocular and monocular feedback conditions for each viewing angle. It can be seen that peak wrist velocities scaled linearly with object distance in all cases, indicating that this aspect of the movements was stereotypical in all conditions (Jeannerod, 1984, 1988).

|  |
| --- |
| **Fig. 5** Peak wrist velocity scaling. Average peak wrist velocity as a function of object distance (collapsed across object size) for (a) 15, (b) 52.5, and (c) 90 deg viewing angles. Solid circles denote binocular feedback and open circles denote monocular feedback. Error bars denote ±1 SEM. |

**PEAK GRIP APERTURE ‘SCALING’**

Figure 6 plots mean peak grip aperture in each condition, as a function of object size (collapsed across distance). The panels plot binocular and monocular feedback conditions at each viewing angle. Peak grip apertures scaled linearly with object size in all cases, in the stereotypical manner (Jeannerod, 1984, 1988).

|  |
| --- |
| **Fig. 6** Peak grip aperture scaling. Average peak grip aperture as a function of object size (collapsed across object distance) for (a) 15, (b) 52.5, and (c) 90 deg viewing angles. Again, solid circles denote binocular feedback and open circles denote monocular feedback. Error bars denote ±1 SEM. |

**ANALYSIS OF MOVEMENT TRAJECTORIES**

Figure 7 plots overall average trajectories of the thumb for each condition, and shows the line-of-sight for the three viewing angles. The data are normalised with respect to the orientation of the table surface (which differed across viewing-angle conditions), and object distance, so that trajectories can be meaningfully compared. Details of the analysis are described in the figure caption. It can be seen that in the latter half of the movements, in particular, reach trajectories were very similar across all conditions. Thus, moving the participants’ viewpoint did alter the viewing angle with respect to the movement direction.

|  |
| --- |
| **Fig. 7** Thumb movement trajectories. The figure plots a side view (i.e. in the median plane) of the overall average spatial trajectories of the thumb in each viewing condition (height of the thumb above the table surface as a function of distance from the start button). The movement data were normalised with respect to the orientation of the table surface, and to object distance. To do this, for each trial we first identified the position of the thumb (*i*) before the movement began, and (*ii*) at the movement end point. We then specified the start point on each trial as 0,0 and the end point as 350,0 (350 mm being the average object distance in the experiment). We then calculated the spatial trajectory of each movement with respect to these two datum points, yielding normalised trajectories. We computed average trajectories within each participant by averaging across all his or her trials within a viewing condition. We then averaged these trajectories *across* participants to produce overall average trajectories, plotted in the figure. Red, green and blue curves denote the 15, 52.5 and 90 deg viewing angles, respectively. Solid lines indicate binocular feedback, and dashed lines indicate monocular feedback. The shaded zones around the binocular 15 deg and binocular 90 deg data denote between-subjects standard errors of the respective trajectories (±1 SEM). To aid legibility we did not plot standard errors for the other conditions, but they were of similar magnitude. The three solid grey lines (with eye icons) show the lines of sight in the three viewing angle conditions. The dark-grey lines (with hand icons) show the average movement directions over the last half (solid line) and last third (dashed line) of the movement. These were calculated as the best-fitting linear regression to the relevant portion of the trajectory data, averaged across all viewing conditions. |

**REFERENCES**

Jeannerod, M. (1984). The timing of natural prehension movements. *Journal of Motor Behavior, 16(3)*, 235–54.

Jeannerod, M. (1988). *The neural and behavioural organization of goal-directed movements*. Oxford: Clarendon Press.
